# The Mechanism of Enantioselective Neurosteroid Actions on GABA_A_ Receptors

**DOI:** 10.3390/biom13020341

**Published:** 2023-02-09

**Authors:** Hiroki Tateiwa, Satyanarayana M. Chintala, Ziwei Chen, Lei Wang, Fatima Amtashar, John Bracamontes, Allison L. Germann, Spencer R. Pierce, Douglas F. Covey, Gustav Akk, Alex S. Evers

**Affiliations:** 1 Department of Anesthesiology, Washington University School of Medicine, St. Louis, MO 63110, USA; 2 Department of Anesthesiology and Intensive Care Medicine, Kochi Medical School, Kochi 7838505, Japan; 3 Taylor Institute for Innovative Psychiatric Research, St. Louis, MO 63110, USA; 4 Department of Anesthesiology, Union Hospital, Tongji Medical College, Huazhong University of Sciences and Technology, Wuhan 430074, China; 5 Department of Psychiatry, Washington University School of Medicine, St. Louis, MO 63110, USA; 6 Department of Developmental Biology (Pharmacology), Washington University School of Medicine, St. Louis, MO 63110, USA

**Keywords:** neurosteroids, GABA-A receptors, enantiomers, photolabeling

## Abstract

The neurosteroid allopregnanolone (ALLO) and pregnanolone (PREG), are equally effective positive allosteric modulators (PAMs) of GABA_A_ receptors. Interestingly, the PAM effects of ALLO are strongly enantioselective, whereas those of PREG are not. This study was aimed at determining the basis for this difference in enantioselectivity. The oocyte electrophysiology studies showed that *ent*-ALLO potentiates GABA-elicited currents in α_1_β_3_ GABA_A_ receptors with lower potency and efficacy than ALLO, PREG or *ent*-PREG. The small PAM effect of *ent*-ALLO was prevented by the α_1_(Q242L) mutation in the intersubunit neurosteroid binding site between the β_3_ and α_1_ subunits. Consistent with this result, neurosteroid analogue photolabeling with mass spectrometric readout, showed that *ent*-ALLO binds weakly to the β_3_-α_1_ intersubunit binding site in comparison to ALLO, PREG and *ent*-PREG. Rigid body docking predicted that *ent*-ALLO binds in the intersubunit site with a preferred orientation 180° different than ALLO, PREG or *ent*-PREG, potentially explaining its weak binding and effect. Photolabeling studies did not identify differences between ALLO and *ent*-ALLO binding to the α_1_ or β_3_ intrasubunit binding sites that also mediate neurosteroid modulation of GABA_A_ receptors. The results demonstrate that differential binding of *ent*-ALLO and *ent*-PREG to the β_3_-α_1_ intersubunit site accounts for the difference in enantioselectivity between ALLO and PREG.

## 1. Introduction

Enantiomers are molecules with opposite stereochemistry at every chiral center. They have identical physical properties, but have non-superimposable mirror image structures and rotate polarized light in opposite directions. Enantiomers of hydrophobic ligands have equal solubility in lipids and are equally effective at disrupting lipid bilayers [1,2,3]. Enantioselectivity has thus been used as a criterion for demonstrating that a hydrophobic ligand modulates membrane protein activity by interacting with specific chiral protein binding sites rather than by perturbing membrane properties [2,4].

The demonstration that the anesthetic neurosteroid allopregnanolone (3α-hydroxy-5α-pregnan-20-one ALLO) is an enantioselective modulator of GABA_A_ receptor currents and an enantiospecific anesthetic provided the initial evidence that neurosteroids bind to specific sites on GABA_A_ receptors to enhance GABA currents and produce anesthesia [5,6]. Pregnanolone (3α-hydroxy-5β-pregnan-20-one; PREG), the 5β-epimer of ALLO, is also a positive allosteric modulator of GABA_A_ currents and an anesthetic, but has minimal enantioselectivity either as a modulator of GABA_A_ receptors or as an anesthetic [7].

It has subsequently been shown using site-directed mutagenesis [8], neurosteroid analogue photoaffinity labeling [9,10,11] and x-ray crystallography [12,13,14] that PAM neurosteroids bind in an intersubunit site between the third transmembrane helix (TM3) of a GABA_A_ β-subunit and the first transmembrane helix (TM1) of an adjacent α-subunit. Occupancy of this site is the major contributor to the PAM effect of neurosteroids and mutations to the Q242 and W246 residues in this site largely ablate PAM activity [8,15,16,17]. Photoaffinity labeling studies have shown that PAM neurosteroid binding to intrasubunit sites in the α-subunit (between TM1 and TM4) and the β-subunit (between TM3 and TM4) also contributes to modulation of GABA_A_ receptors [9,15,16,18].

To determine the molecular mechanism underlying the differential enantioselective effects of ALLO and PREG, we examined the binding of ALLO, PREG and their enantiomers (*ent*-ALLO and *ent*-PREG) to the three neurosteroid binding sites on α_1_β_3_ GABA_A_ receptors. Site-specific binding was determined by measuring the ability of the PAM neurosteroids and their enantiomers to prevent neurosteroid analogue photolabeling of peptides in each of the binding sites. We also used KK152, the enantiomer of the ALLO-analogue photolabeling reagent KK123, to confirm enantiomer binding to the intrasubunit sites. The binding results were correlated with electrophysiological studies examining the effects of the PAM neurosteroids and their enantiomers on GABA_A_ currents in wild type receptors and in receptors with an α_1_(Q242L) mutation in the intersubunit binding site. Collectively, the experimental data demonstrate that the difference in enantioselectivity between ALLO and PREG results from the differential binding of *ent*-ALLO and *ent*-PREG in the canonical β_3_-α_1_ intersubunit binding site. The structural basis for this difference was explored using rigid body molecular docking.

## 2. Materials and Methods

### 2.1. Receptor Expression in Xenopus laevis Oocytes and Electrophysiological Recordings

GABA_A_ receptors were expressed in oocytes from the African clawed frog (*X. laevis*). The oocytes were purchased as quarter ovaries from Xenopus1 (Dexter, MI, USA). The ovaries were digested in a 2% *w*/*v* (mg/mL) solution of collagenase A in ND96 solution (96 mM NaCl, 2 mM KCl, 1.8 mM CaCl_2_, 1 mM MgCl_2_, 5 mM HEPES; pH 7.4) containing 100 U/mL penicillin and 100 μg/mL streptomycin for 30 to 40 min at 37 °C.

The cDNAs containing the human α_1_ or β_3_ subunits were linearized with XbaI (NEB Labs, Ipswich, MA, USA), and the cRNAs were generated using T7 mMessage mMachine (Ambion, Austin, TX, USA). The α_1_(Q242L) and α_1_(V227W) mutations were generated using the QuikChange Site-Directed Mutagenesis Kit (Agilent Technologies, Santa Clara, CA, USA), and the coding region was fully sequenced prior to use. The functional properties of the α_1_(Q242L) and α_1_(V227W) mutants have been reported in previous publications [8,9,16,17]. The oocytes were injected with a total of 12 ng cRNA. The ratio of cRNAs was 5:1 (α_1_:β_3_) to minimize the expression of β_3_ homomeric receptors. Following injection, the oocytes were incubated in ND96 at 16 °C for 2 days prior to conducting electrophysiological recordings.

**Figure 1:**
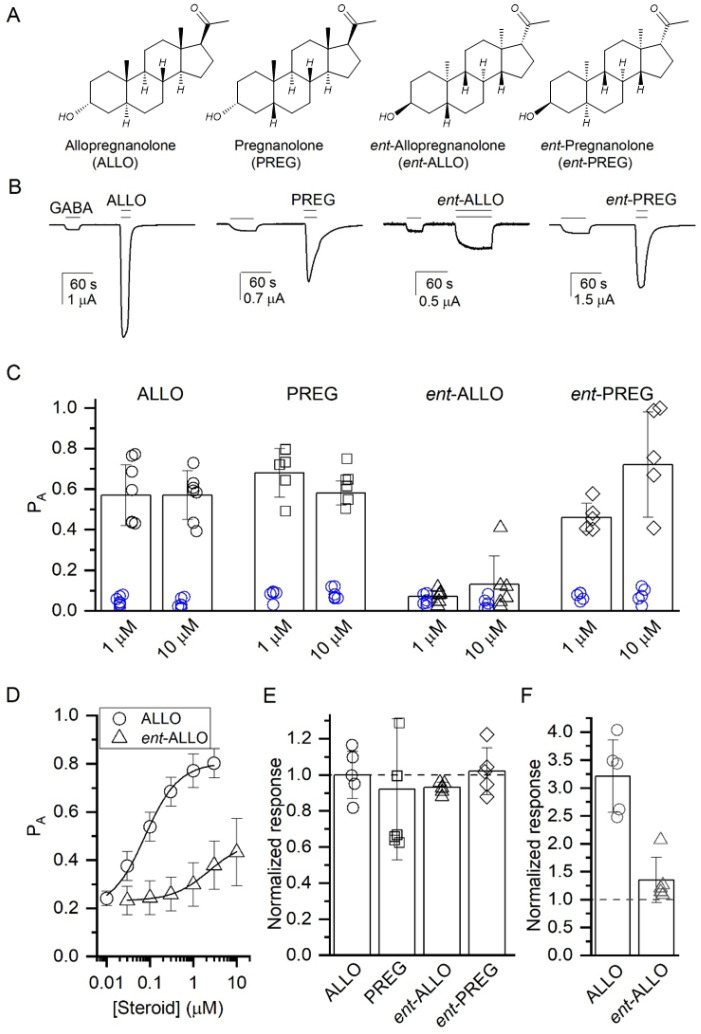
Effects of ALLO, PREG, *ent*-ALLO and *ent*-PREG on GABA-elicited currents in α_1_β_3_ GABA_A_ receptors expressed in *Xenopus* oocytes. (**A**) Steroid structures (**B**) Representative traces of currents elicited by low GABA (0.2–0.5 µM; P_A_ = 0.06 ± 0.03) in the presence or absence of 10 μM concentrations of steroids. (**C**) Enhancement of currents elicited with low GABA (n = 5) by 1 and 10 μM steroids. Blue symbols indicate response with GABA alone and black symbols are for GABA + steroid. Open probabilities for steroid-enhanced currents at 1 and 10 μM were compared using one way ANOVA. There was a statistically significant difference between groups as determined by one-way ANOVA (*F*(3,20) = 33.29, *p* < 0.001 for 1 µM; F(3,20) = 18.45, *p* < 0.001 for 10 µM). A Bonferroni post-hoc multiple comparison test of the means revealed that at both 1 and 10 µM the effect of ent-ALLO was significantly different compared to the effects of ALLO, PREG, and *ent*-PREG (*p* < 0.001 for each comparison). (**D**) Concentration-response curves comparing potentiation of GABA-elicited currents (GABA = 0.8–3 µM; target Pa = 0.2) by ALLO and *ent*-ALLO. Fitting to an MWC model (Equation (1)) indicates that *ent*-ALLO is both less potent than ALLO (K_R,steroid_ values of 0.22 ± 0.19 and 2.39 ± 1.17 µM, respectively) and less efficacious (c values of 0.199 ± 0.053 and 0.561 ± 0.065, respectively). (**E**) Potentiation of GABA (target P_A_ = 0.06) by ALLO, *ent*-ALLO, PREG and *ent*-PREG in α_1_(Q242L)β_3_ receptors. The data indicate that potentiation of GABA responses by all of the steroids is eliminated by a mutation in the intersubunit site. (**F**) Potentiation of GABA (P_A_ < 0.1) by ALLO and *ent*-ALLO (10 µM) in α_1_(V227W)β_3_ receptors. The difference in efficacy between ALLO and ent-ALLO is preserved in the V227 W mutant. Figures (**E**,**F**) show the ratio of the response to GABA + steroid to GABA alone, with a ratio of 1 indicating no steroid response.

Electrophysiological recordings were conducted using standard two-electrode voltage clamp. Borosilicate capillary glass tubing (G120F-4, OD = 1.20 mm, ID = 0.69 mm; Warner Instruments, Hamden, CT, USA) was used for voltage and current electrodes. The electrodes were filled with 3 M KCl and had resistances of 0.3–1 MΩ. The oocytes were clamped at −60 mV. The chamber (RC-1Z; Warner Instruments) was perfused with ND96 at 5–8 mL min^−1^. Solutions were gravity-applied from 30-mL glass syringes with glass luer slips via Teflon tubing, to minimize drug absorption. The current responses were amplified with an OC-725C amplifier (Warner Instruments), digitized with a Digidata 1200 series digitizer (Molecular Devices, San Jose, CA, USA) and stored using pClamp (Molecular Devices). The peak amplitude was determined using Clampfit (Molecular Devices). The stock solution of GABA was made in ND96 bath solution at 500 mM, stored in aliquots at −20 °C, and diluted as needed on the day of experiment. Activation by steroids was tested by co-applying a steroid with 0.2–0.5 µM GABA for wild type receptors (Figure 1B,C) and 0.5–3.0 µM GABA for the α_1_(Q242L)β_3_ receptors (Figure 1E) to achieve a target probability of being in the active state (P_A_) of 0.05–0.1. For steroid concentration-response curves (Figure 1D), steroid was co-applied with 0.8–3 µM GABA to achieve a target P_A_ of ~0.2. The steroids were dissolved in DMSO at 10 mM and stored at room temperature.

The effects of steroids were estimated by calculating the ratio of the peak responses to GABA + steroid and GABA alone. Descriptive analysis of steroid-potentiation was carried out by fitting the steroid concentration-response data to the Hill equation. Mechanistic analysis of steroid-potentiation was conducted in the framework of a cyclic two-state (Resting-Active) concerted transition model [19,20]. In brief, the raw current amplitudes were converted to units of P_A_ by normalizing the responses to GABA or GABA + steroid to the response to 1 mM GABA + 50 µM propofol that was considered to generate a response with peak P_A_ of ~1 [21]. The concentration response data were then fitted to the state function:

(1) $$P_{A}=\frac{1}{1+L*{\left[\frac{1+[\mathrm{steroid}]/K_{R,\mathrm{steroid}}}{1+[\mathrm{steroid}]/(K_{R,\mathrm{steroid}}c_{\mathrm{steroid}})}\right]}^{N_{\mathrm{steroid}}}}$$

where L* is the P_A_ of the response to GABA alone in the same cell and is calculated as (1 − P_A,GABA_)/P_A,GABA_, [steroid] is the concentration of steroid, K_R,steroid_ is the equilibrium dissociation constant of the steroid in the resting receptor, *c*_steroid_ is the ratio of the equilibrium dissociation constant in the active receptor to K_R,steroid_, and N_steroid_ is the number of steroid binding sites (by convention, constrained to 2). A higher value of *c*_steroid_ indicates lower efficacy. Free energy change provided by the steroid can be calculated as ∆G = NRT × ln(*c*_steroid_). The curve-fitting was carried out using Origin 2020 (OriginLab Corp, Northampton, MA, USA). The data are presented as mean ± SD.

### 2.2. Cell Culture, Protein Expression and Membrane Preparation

A tetracycline-inducible cell line expressing human α_1_-8xHis-FLAG and human β_3_ GABA_A_ receptor subunits in HEK-T-Rex™-293 cells was generated and propagated as previously described [9]. Briefly, stably transfected cells were cultured under the following conditions: cells were maintained in DMEM/F-12 50/50 medium containing 10% fetal bovine serum (tetracycline-free, Takara, Mountain View, CA, USA), penicillin (100 units/mL), streptomycin (100 g/mL), blasticidin (2 mg/mL), hygromycin (50 µg/mL) and zeocin (20 µg/mL) at 37 °C in a humidified atmosphere containing 5% CO_2_. Cells were passaged twice each week, maintaining subconfluent cultures. For protein production, cells were plated into dishes. When the cells reached 50% confluence, GABA receptor expression was induced with 1 µg/mL doxycycline with the addition of 5 mM sodium butyrate and the cells were grown to confluence. 48 to 72 h after induction the cells were harvested and washed twice with a buffer containing 10 mM potassium phosphate, 100 mM potassium chloride (pH 7.5) plus protease inhibitors (Sigma-Aldrich, St. Louis, MO, USA). Cells were collected by centrifugation at 1000× *g* at 4 °C for 5 min and homogenized with a glass mortar and a Teflon pestle for ten strokes on ice. Membranes were collected by centrifugation at 40,000× *g* at 4 °C for 30 min and resuspended in a buffer containing 10 mM potassium phosphate, 100 mM potassium chloride (pH 7.5). Protein concentration was determined with micro-BCA protein assay (Thermo Fisher Scientific, Waltham, MA, USA). GABA_A_ receptor content of the membranes was determined by measuring the B_max_ of [^3^H]muscimol binding as previously described [9] and assuming that each mole of receptor contains two muscimol binding sites. Membranes were stored at −80 °C.

### 2.3. Photolabeling and Purification of α_1_β_3_ GABA_A_R

The syntheses of the neurosteroid photolabeling reagents KK123, KK152 and KK200 are detailed in previous reports [22,23]. For each photolabeling experiment, 10–20 mg of HEK cell membrane protein containing 100–150 pmoles of α_1_β_3_ GABA_A_ receptor was used. Frozen membranes were thawed and resuspended at a final concentration of 1.25 mg ml^−1^ in a buffer containing 10 mM potassium phosphate, 100 mM potassium chloride (pH 7.5), and 1 mM GABA. For the photolabeling competition experiments, 3 μM KK123 or KK200 in the presence of 30 μM competitor (ALLO, *ent*-ALLO, PREG, and *ent*-PREG), or the same volume of ethanol for control group, was added to the membrane suspension and incubated on ice for 1 h. The samples were then irradiated in a quartz cuvette for 5 min, using a photoreactor emitting light with wavelengths >320 nm [24]. Membranes were then collected by centrifugation at 20,000× *g* at 4 °C for 30 min. The photolabeled membrane proteins were resuspended in lysis buffer containing 1% n-dodecyl-β-D-maltoside (DDM) (Anatrace, Maumee, OH, USA), 0.25% cholesteryl hemisuccinate (CHS) (Anatrace, Maumee, OH, USA), 50 mM Tris (pH 7.5), 150 mM NaCl, 2 mM CaCl_2_, 5 mM KCl, 5 mM MgCl_2_, 1 mM EDTA, 10% glycerol at a final concentration of 1 mg ml^−1^. The membrane protein suspension was homogenized using a glass mortar and a Teflon pestle and incubated at 4 °C overnight. The protein lysate was centrifuged at 15,000× *g* at 4 °C for 30 min and the supernatant was incubated with 0.5 mL anti-FLAG agarose (Sigma-Aldrich, St. Louis, MO, USA) at 4 °C for 2 h. The anti-FLAG agarose was then transferred to an empty column, followed by washing with 20 mL of washing buffer (50 mM triethylammonium bicarbonate and 0.02% DDM). GABA_A_ receptors were eluted with aliquots of 200 μg mL^−1^ FLAG tag peptide and 100 μg ml^−1^ 3X FLAG (ApexBio) in the washing buffer. Pooled eluates (4 mL) containing GABA_A_ receptors were concentrated to 100 μL using 100 kDa cut-off centrifugal filters.

### 2.4. Middle-Down MS Analysis

The purified α_1_β_3_ GABA_A_ receptors (100 μL) were reduced with 5 mM tris(2-carboxyethyl)phosphine for 30 min, followed by alkylation with 5 mM N-ethylmaleimide (NEM) for 45 min in the dark. The NEM was quenched with 5 mM dithiothreitol (DTT) for 15 min. These three steps were carried out at room temperature. Samples were then digested with 8 μg of trypsin for 7 days at 4 °C to obtain maximal recovery of TMD peptides. The digestions were terminated by adding formic acid to a final concentration of 1%, followed directly by LC-MS analysis on an Orbitrap Elite mass spectrometer. 20 μL samples were injected into a home-packed PLRP-S (Agilent, Santa Clara, CA, USA) column (10 cm × 75 μm, 300 Å), separated with a 135 min gradient from 10% to 90% acetonitrile, and introduced to the mass spectrometer at 800 nL min^−1^ with a nanospray source. MS acquisition was set as a MS1 Orbitrap scan (resolution of 60,000) followed by top 20 MS2 Orbitrap scans (resolution of 15,000) using data-dependent acquisition, and exclusion of singly charged precursors. Fragmentation was performed using high-energy dissociation with normalized energy of 35%. Analysis of datasets was performed using Xcalibur (Thermo Fisher Scientific) to manually search for TM1, TM2, TM3 or TM4 tryptic peptides with or without neurosteroid photolabeling modifications. Photolabeling efficiency was estimated by generating extracted chromatograms of unlabeled and labeled peptides, determining the area under the curve, and calculating the abundance of labeled peptide/(unlabeled + labeled peptide). Analysis of statistical significance comparing the photolabeling efficiency of KK123 and KK200 for α_1_β_3_ GABA_A_R was determined using one-way ANOVA with Bonferroni’s multiple comparisons test (GraphPad Prism version 9.4.0 for Windows, GraphPad Software, San Diego, CA, USA). MS2 spectra of photolabeled TMD peptides were analyzed by manual assignment of fragment ions with and without photolabeling modification. Fragment ions were accepted based on the presence of a monoisotopic mass within 20 ppm mass accuracy. In addition to manual analysis, PEAKS (Bioinformatics Solutions Inc., Waterloo, ON, Canada) database searches were performed for datasets of photolabeled α_1_β_3_ GABA_A_R. Search parameters were set for a precursor mass accuracy of 20 ppm, fragment ion accuracy of 0.1 Da, up to three missed cleavages on either end of the peptide, false discovery rate of 0.1%, and variable modifications of methionine oxidation, cysteine alkylation with NEM and DTT, and neurosteroid analogue photolabeling reagents on any amino acid.

### 2.5. Molecular Docking and Binding Energy Calculations

The molecular coordinates of ALLO (PubChem CID: 92786) and PREG (PubChem CID: 31402) were obtained from PubChem. The structures of *ent*-ALLO and *ent*-PREG were generated by inverting the chiral configurations of all the chiral centers in the structures of ALLO and PREG. These structures were then energy minimized using UFF force field and Steepest Descent algorithm in the Avogadro software [25] to obtain the coordinates of *ent*-ALLO and *ent*-PREG. The docking template of α_1_β_3_γ_2_ was generated using the CryoEM structure (PDB: 6HUO) [26]. The ligands bound to the protein were deleted and the structure was energy minimized in Chimera ver. 1.16 [27]. DockPrep was used to add hydrogens and charges to the protein structure. The interface between β_3_-TM3 and α_1_-TM1 was used for grid generation of size 20 × 18 × 29 Å encompassing the neurosteroid binding site. Docking was performed using AutoDock Vina [28] in the Chimera software to obtain the binding energies and binding poses of ALLO, PREG, *ent*-ALLO and *ent*-PREG.

## 3. Results

### 3.1. Potentiation of α_1_β_3_ GABA_A_ Currents by Enantiomeric Neurosteroids

We initially examined the effects of ALLO, *ent*-ALLO, PREG and *ent*-PREG (Figure 1A) on GABA-elicited currents in wild-type α_1_β_3_ GABA_A_ receptors expressed in Xenopus oocytes. Representative traces (Figure 1B) illustrate that 10 µM concentrations of each of the steroids potentiate the currents elicited by low (0.2–0.5 µM; P_A_ = 0.06 ± 0.03; mean ± S.D. from 48 cells) GABA. Quantitative analysis (Figure 1C) shows that 10 µM ALLO, PREG and *ent*-PREG enhance GABA-elicited currents to a similar extent (*p* > 0.05). In contrast, *ent*-ALLO enhances GABA-elicited currents to a lesser extent than any of the other steroids (*p* < 0.001 vs. ALLO, PREG and *ent*-PREG).

Concentration-response curves (Figure 1D) comparing the potentiation of GABA-elicited currents (GABA = 0.8–3 µM; target P_A_ = 0.2) indicate lower apparent potency for *ent*-ALLO (EC_50_ = 2.00 ± 0.30 µM; n = 5) than ALLO (EC_50_ = 0.08 ± 0.01 µM; n = 5). Fitting the data from Figure 1D to Equation (1) yielded K_R,steroid_ values of 0.22 ± 0.19 and 2.39 ± 1.17 µM and *c*_steroid_ values of 0.199 ± 0.053 and 0.561 ± 0.065 for ALLO and *ent*-ALLO, respectively, indicating that *ent*-ALLO is both less potent and less efficacious than ALLO.

To determine whether the modest PAM effect of *ent*-ALLO is mediated by the β_3_/α_1_ intersubunit binding site, we next examined the effects of the neurosteroid enantiomeric pairs in receptors with an α_1_(Q242L) mutation in the intersubunit site. The enhancement of GABA_A_ currents by ALLO, PREG and their enantiomers was eliminated in α_1_(Q242L)β_3_ receptors. This is quantitatively illustrated in Figure 1E, which shows the ratio of the effect of a 10 µM concentration of each steroid to the baseline GABA response. These data indicate that while *ent*-ALLO is a weak allosteric agonist, its PAM effect is still predominantly mediated by the canonical β_3_/α_1_ intersubunit binding site.

PAM actions of steroids are additionally mediated by the α_1_-intrasubunit site [9,16]. To confirm the lower efficacy of *ent*-ALLO at the β_3_/α_1_ intersubunit site, we compared the effects of 10 µM ALLO and *ent*-ALLO on currents elicited by GABA (P_A_ < 0.1) in receptors in which the actions of steroids in the α_1_-intrasubunit site are prevented by the α_1_(V227W) mutation. In the α_1_(V227W)β_3_ receptor, application of ALLO potentiated the response to GABA to 321 ± 65% (n = 5) of control while application of *ent*-ALLO potentiated to only 135 ± 41% (n = 5) of control (Figure 1F). These data confirm that the difference in efficacy between ALLO and *ent*-ALLO acting at the β_3_/α_1_ intersubunit binding site accounts for the enantioselective PAM effect of ALLO. We note that the effects of mutations to the β_3_/α_1_ intersubunit site and the α_1_-intrasubunit site (α_1_(Q242L) and α_1_(V227W), respectively) are not strictly additive, indicating that the two sites are allosterically linked in the α_1_β_3_ receptor.

### 3.2. Competitive Prevention of Labeling with ALLO-Analogue Photolabeling Reagents

**Figure 2:**
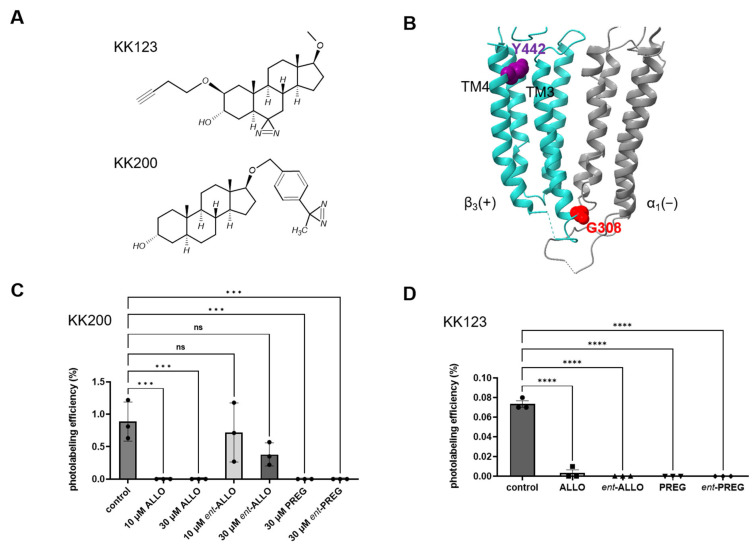
Competitive inhibition of neurosteroid analogue photolabeling. (**A**) Structures of the neurosteroid analogue photolabeling reagents KK200 and KK123. (**B**) Structure of the α_1_β_3_ GABA_A_ receptor transmembrane domains highlighting residue Y442 on β_3_-TM4 (purple) which is labeled by KK123 and residue G308 on β_3_-TM3 (red) which is labeled by KK200. The α_1_ subunit is shown in grey and the β_3_ subunit in turquoise. (**C**) photolabeling efficiency of β_3_ subunit TM3 by 3 μM KK200 in α_1_β_3_ GABA_A_ receptors in the absence (control) or presence of either 10 or 30 μM allopregnanolone (ALLO), *ent*-allopregnanolone (*ent*-ALLO), pregnanolone (PREG) or *ent*-pregnanolone (*ent*-PREG). There was a statistically significant difference between groups as determined by one-way ANOVA (F(6, 14) = 9.393, *p* < 0.001). Bonferroni’s post-hoc multiple comparison test of the means showed that the effects of ALLO, PREG, and *ent*-PREG were all significantly different than control (*p* < 0.001 vs. control for each comparison) whereas the effects of *ent*-ALLO were not significantly different than control (*p* = 0.85 for control vs. 10 µM *ent*-ALLO and *p* = 0.06 for control vs. 30 µM *ent*-ALLO). (**D**) Photolabeling efficiency of β_3_ subunit TM4 by 3 μM KK123 in α_1_β_3_ GABA_A_ receptors in the absence (control) or presence of 30 μM competitive steroid. There was a statistically significant difference between groups as determined by one-way ANOVA F(4, 10) = 237.0, *p* < 0.0001). Bonferroni’s post-hoc multiple comparison test of the means showed that the effects of ALLO, PREG, *ent*-ALLO and *ent*-PREG were all significantly different than control (*p* < 0.0001 vs. control for each comparison) In Panels C and D data are shown as mean ± range with n =3 for each point. Statistical differences: *** = *p* < 0.001; **** = *p* < 0.0001; ns = not significant.

To directly determine whether ALLO, PREG and their enantiomers bind to each of the neurosteroid binding sites on α_1_β_3_ GABA_A_ receptors, we measured their ability to prevent neurosteroid-analogue photolabeling of peptides in each of these sites. It has previously been shown that the ALLO-analogue photolabeling reagent, KK200 (Figure 2A), labels residue G308 at the cytoplasmic end of β_3_ TM3 in the interface between the β_3_ and α_1_ subunits (Figure 2B and Figure S1). [9]. The ability of each neurosteroid to prevent photolabeling of G308 by 3 µM KK200 was used to assay binding to the β_3_/α_1_ intersubunit site. 30 µM ALLO, PREG and *ent*-PREG completely prevented KK200 labeling of the β_3_ TM3 peptide (*p* < 0.001 vs. control), whereas 30 µM *ent*-ALLO partially, but not significantly (*p* = 0.06, control vs. 30 μM *ent*-AlloP), reduced labeling. 10 µM ALLO also completely inhibited KK200 labeling of G308, whereas 10 µM *ent*-ALLO did not inhibit labeling, confirming that ALLO binds enantioselectively in the intersubunit site (Figure 2C). While there was no significant difference between the photolabeling efficiency of the control and the 10 or 30 μM *ent*-ALLO samples, the trend toward reduced labeling with increasing concentrations of *ent*-ALLO suggests that it binds to the intersubunit site with very low affinity. Of note, KK200 also labels the α_1_ intrasubunit site at residue N408 on α_1_-TM4, but the efficiency of labeling was insufficient to measure competitive prevention of photolabeling.

### 3.3. Docking Simulation

**Figure 3:**
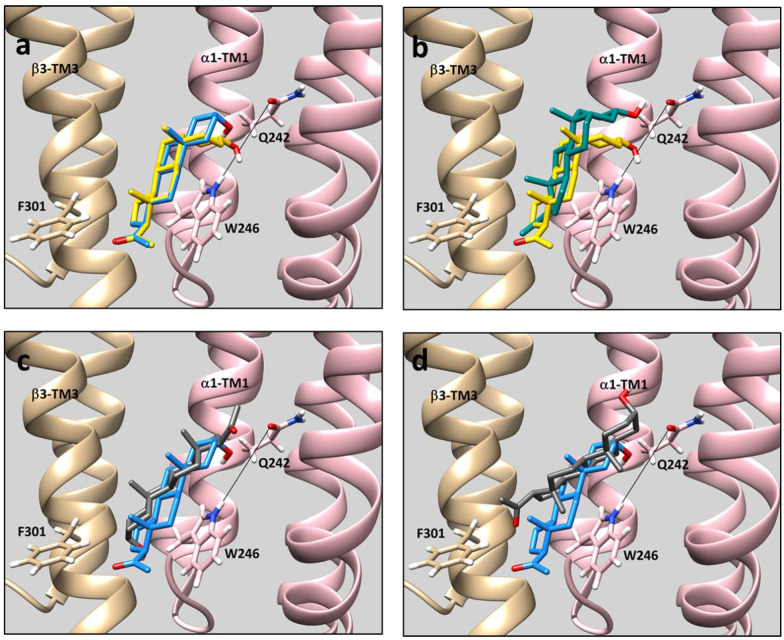
Comparison of steroid docking poses in the β_3_/α_1_ intersubunit binding site of the GABA_A_ receptor using the cryo-EM structure (PDB 6HUO) of a α_1_β_3_γ_2_ GABA_A_ receptor. The β_3_ subunit is shown in tan and the α_1_ subunit in pink. Sidechains of the α_1_(Q242) and α_1_(W246) residues are shown connected by a line indicating the axis of the coordinated hydrogen bonds formed by these two residues with the steroid 3-hydroxy group. The side chain of β_3_(F301), a residue known to line the steroid binding site is also shown. The structure of ALLO is shown in blue, *ent*-ALLO in grey, PREG in yellow and *ent*-PREG in green. (**a**) ALLO and PREG in their lowest energy poses. (Both steroids are in Orientation #1 (See Table 1)). The steroid rings, methyl groups and methyl ketones are aligned with the 3-hydroxy groups of both steroids positioned to form hydrogen bonds with Q242 and W246. (**b**) PREG and *ent*-PREG in their lowest energy poses. (Both steroids are in Orientation #1.) The methyl groups and methyl ketones of both steroids are similarly oriented and the 3-hydroxy groups of both steroids are positioned to hydrogen bond with Q242. (**c**) ALLO and *ent*-ALLO in their lowest energy poses. While the methyl groups of the two steroids are similarly positioned, the 3-hydroxyl group of *ent*-ALLO is oriented 180° from ALLO and points to F301; it is thus not positioned to form a hydrogen bond with Q242 or W246. (ALLO is in Orientation #1, whereas *ent*-ALLO is in Orientation #2.) (**d**) Comparison of ALLO (lowest energy pose) with *ent*-ALLO in the lowest energy pose in which the 3-hydroxy group points toward the α_1_ subunit. (Both steroids are in Orientation #1.) The methyl groups of the two steroids are oriented in opposite directions and the 3-hydroxy group of *ent*-ALLO is pointing away from Q242. The absence of a hydrogen bond and the malposition of the methyl groups likely makes this an energetically unfavorable pose for *ent*-ALLO binding. The Vina docking scores for the steroids in each pose are shown in Table 1.

To probe the structural basis for the reduced binding of *ent*-ALLO to the intersubunit site, rigid body docking of ALLO, *ent*-ALLO, PREG and *ent*-PREG to the β_3_/α_1_ intersubunit site was analyzed using a cryo-EM structure (PDB: 6HUO) of an α_1_β_3_γ_2_ GABA_A_ receptor [26]. The neurosteroids docked between β_3_-TM3 and α_1_-TM1 in poses with two distinct orientations: (1) poses in which the steroid 3-hydroxy group is proximal to the Q242 residue on α_1_–TM1 and the 17-methyl ketone is proximal to F301 on β_3_-TM3 (orientation #1) or; (2) poses in which the 3-hydroxy group is proximal to F301 on β_3_-TM3 and the 17-methyl ketone is near Q242 on α_1_TM1 (orientation #2). For ALLO, the lowest energy pose is in orientation #1 with the 18 and 19 methyl groups both pointing to the lipid surface of β_3_-TM3 (Figure 3A). This is the same pose observed in the X-ray structures of THDOC bound to the GLIC-α_1_GABA_A_ chimera [13] and alphaxalone bound to the ELIC-α_1_GABA_A_ chimera [14]. ALLO was also observed in poses with orientation #2, but with significantly less negative binding energy (Table 1). The energetically preferred docking pose for PREG is also in orientation #1 and is almost identical to the pose observed in the crystal structure of PREG bound to the β_3_/α_5_ chimeric GABA_A_ receptor [12] (Figure 3a). In the preferred poses of ALLO and PREG docked to the β_3_/α_1_ intersubunit site, their 3-hydroxy, 18 and 19 methyl and methyl ketone groups all assume overlapping positions, consistent with their similar potency and efficacy as GABA_A_ receptor PAMs. *ent*-PREG docks with similar energy in either orientation #1 or #2 (Table 1). A comparison of *ent*-PREG and PREG in their lowest energy poses (both orientation #1) shows that their methyl ketones and 18 and 19 methyl groups roughly align and their 3-hydroxy groups both point toward α_1_-TM1, consistent with the minimal enantioselectivity of PREG (Figure 3b). In contrast to the other neurosteroids, the energetically preferred pose of *ent*-ALLO is in orientation #2. In this pose, the C18 and C19 methyl groups are aligned with the ALLO methyl groups, but the 3-hydroxy group is not positioned to form a hydrogen bond with either Q242 or W246 (Figure 3c). *ent*-ALLO docking in orientation #1 is less energetically favorable (Table 1). A comparison of ALLO to *ent*-ALLO binding in orientation #1 shows that the methyl groups of the two steroids point in almost opposite directions and the *ent*-ALLO 3-hydroxy group is not proximal to Q242. (Figure 3d).

### 3.4. Steroid Binding to the α_1_ and β_3_ Intrasubunit Site on α_1_β_3_ GABA_A_ Receptors

While electrophysiological and mutational data indicate that the PAM effects of ALLO, *ent*-ALLO, PREG and *ent*-PREG are largely mediated by the β_3_/α_1_ intersubunit site, neurosteroids also bind to and act through β_3_ and α_1_ intrasubunit sites [9,16]. To examine binding to the β_3_ intrasubunit site, we measured the ability of the ALLO and PREG enantiomeric pairs to prevent photolabeling by the ALLO-analogue photolabeling reagent, KK123 (Figure 2A). KK123 labels residue Y442 on the β_3_-TM4 peptide in the β_3_ intrasubunit site (Figure 2B and Figure S1) [9]. KK123 (3 µM) photolabeling in the β_3_ intrasubunit site was completely prevented by 30 µM ALLO, *ent*-ALLO, PREG, and *ent*-PREG (*p* < 0.001, vs. control, respectively, n = 3 per group) (Figure 2D). KK123 also labeled the α_1_ intrasubunit site at residue Y415 on α_1_-TM4 [9], but the efficiency of labeling was too low to reliably measure competitive prevention of photolabeling.

**Figure 4:**
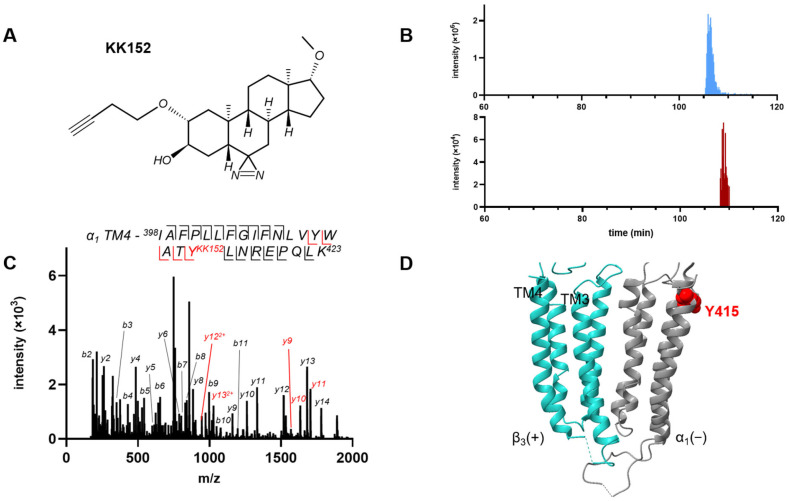
Photolabeling of α_1_β_3_ GABA_A_ receptors by KK152 (the enantiomer of KK123). (**A**) The structure of KK152. (**B**) Extracted ion chromatograms from mass spectrometric analysis illustrating the labeling of α_1_-TM4 peptide in α_1_β_3_ GABA_A_ receptors by KK152. The y-axis shows the intensity of the unlabeled TM4 peptide (top panel) and the KK152-labeled TM4 peptide (bottom panel). The x-axis shows the chromatographic retention time of the unlabeled TM4 peptide (blue, 105.9 min) and the KK152-labeled TM4 peptide (red, 108.5 min) illustrating that the increased hydrophobicity of the labeled peptide lengthens retention time on reversed phase chromatography. (**C**) HCD fragmentation spectrum of the α_1_ subunit TM4 tryptic peptide photolabeled by 30 μM KK152. Red and black indicate fragment ions that do or do not contain KK152, respectively. The peptide sequence shows that the y-ion series of fragment ions contains diagnostic peptides indicating labeling of residue α_1_ (Y415). (**D**) Structure of the α_1_β_3_ GABA_A_ receptor showing the location of Y415, the residue labeled by KK152 at the exoplasmic end of α_1_ TM4. The α_1_ subunit is shown in grey and the β_3_ subunit in turquoise.

Since we could not measure *ent*-ALLO binding to the α_1_ intrasubunit site using competitive prevention of photolabeling, we took an alternative approach of labeling GABA_A_ receptors with KK152, the enantiomer of KK123 (Figure 4A), and identifying the adducted residue using mass spectrometry. KK152 shows minimal GABA_A_ receptor PAM activity, whereas KK123 is a strong GABA potentiator mimicking the effects of the ALLO/*ent*-ALLO enantiomeric pair [23]. We labeled HEK cell membranes containing α_1His/FLAG_β_3_ GABA_A_ receptors with 30 µM KK152, purified the receptors using FLAG-agarose affinity chromatography, and analyzed the protein sequence using middle-down mass spectrometry. Peptides for each of the 8 TMDs (TMD1-4 for α_1_ and β_3_) were identified with 100% peptide-level coverage (Supplementary Figure S2). To identify KK152-labeled peptides, we searched for TMD peptides with the precise add weight of KK152 and with a chromatographic retention time slightly longer than the unmodified peptide. This latter criterion reflects the fact that addition of a steroid increases the hydrophobicity of TMD peptides, shifting their reverse-phase chromatographic retention to later times. Using these criteria, MS1 features corresponding to an α_1_-TM4 peptide with a single KK152 adduct were identified. Extracted ion chromatograms of unlabeled and KK152-labeled α_1_ subunit TM4 peptides are shown in Figure 4B. The blue peak represents the unlabeled peptide with a retention time of 105.9 min and the red peak is the KK152-labeled peptide with a retention time of 108.5 min. The α_1_-TM4 KK152 adducted peptide was identified as ^398^IAFPLLFGIFNLVYWATYLNREPQLK^423^ + KK152 (*m/z* = 1167.0008, *z* = 3). A fragmentation ion spectrum of this peptide identified Tyr415 as the site of KK152 adduction (Figure 4C,D). This is the same residue that is labeled by KK123. While this demonstrates binding of KK152 to the α_1_ intrasubunit site, the photolabeling efficiency observed with 3 µM KK152 (maximum concentration for competition studies) was inadequate to examine competitive prevention of photolabeling.

## 4. Discussion

In this study we determined the molecular basis for the enantioselective action of ALLO and the relative lack of enantioselectivity of PREG as GABA_A_-PAMs. The data show that the enantioselectivity of ALLO is based on differential binding affinity and efficacy of the ALLO enantiomers in the β_3_/α_1_ intersubunit neurosteroid binding site on GABA_A_ receptors. The evidence in support of this conclusion includes: (1) Electrophysiological concentration-response data showing that *ent*-ALLO is at least 20-fold less potent and less efficacious than ALLO as a GABA_A_-PAM; (2) Mutagenesis data showing that the modest PAM effects of *ent*-ALLO are prevented by a mutation (α_1_(Q242L)) known to prevent neurosteroid action in the β_3_/α_1_ intersubunit site [8]; and (3) Photolabeling/mass spectrometry results showing that both *ent*-ALLO and ALLO bind to the α_1_ and β_3_ intrasubunit neurosteroid binding sites, but that *ent*-ALLO binds with very low affinity to the β_3_/α_1_ intersubunit site.

These data also explain the lack of diasteroselectivity between ALLO and its 5β-epimer PREG and the minimal enantioselectivity of PREG. No significant differences in binding to the intrasubunit or intersubunit neurosteroid binding sites were observed between ALLO, PREG and *ent*-PREG. All three compounds had equal efficacy as GABA-PAMs and their PAM effects were prevented by the α_1_(Q242L) mutation, indicating that their PAM actions are largely mediated by binding to the intersubunit site.

Rigid body docking provides some insight into the complex pattern of diastereoselective and enantioselective binding and GABA_A_-PAM activity of the ALLO and PREG enantiomeric pairs. Despite the marked difference in configuration between ALLO and PREG (*cis* vs. *trans* A,B-ring fusion), their energetically preferred poses in the intersubunit site are closely aligned, with their 18 and 19 methyl (“rough surface”) and 17-methyl ketone groups superimposed and their 3-hydroxy groups positioned to form coordinated hydrogen bonds with α_1_(Q242) and α_1_(W246) (Figure 3a) [12]. This explains their lack of diasteroselective GABA_A_-PAM activity. *ent*-PREG, in its preferred pose, is rotated 180° on its short axis in comparison to PREG. In this pose the methyl groups and methyl ketone groups of *ent*-PREG and PREG are aligned and the 3-hydroxy group of *ent*-PREG is positioned to form a hydrogen bond with α_1_(Q242) (Figure 3b). These poses explain the modest degree of enantioselectivity of PREG as a GABA_A_-PAM. In contrast, *ent*-ALLO in its lowest energy pose, is rotated 180° on its long axis in comparison to ALLO, PREG and *ent*-PREG. While the methyl groups of ALLO and *ent*-ALLO align in this pose, the *ent*-ALLO 3-hydroxy group is proximal to β_3_(F301), and is not positioned to form a hydrogen bond with either α_1_(Q242) or α_1_(W246) (Figure 3c). The lowest energy pose in which the 3-hydroxyl group of *ent*-ALLO points toward α_1_(Q242) (Figure 3d) is energetically unfavorable both because the 3-hydroxy group is not positioned to form a hydrogen bond with Q242 and/or W246 and because the 18 and 19 methyl groups point toward W246, potentially interfering with steroid ring-tryptophan stacking interactions.

While docking studies provide inference about binding, the β_3_-α_1_ intersubunit site is on the protein surface and bound neurosteroids interact with both protein and surrounding membrane lipid. The contribution of hydrogen binding to total binding energy is likely to be greater in the nonpolar environment of the lipid membrane than in the *in vacuo* conditions modeled in docking algorithms [29]. As such, the absence of *ent*-ALLO hydrogen bonding to Q242 or W246 may reduce binding energy more than is predicted by docking studies. It is also not clear if *ent*-ALLO dominantly binds in orientation #1 (Figure 3d), orientation #2 (Figure 3c) or both. However, the absence of hydrogen bonding in either orientation is likely to contribute to low affinity and low efficacy.

The finding that *ent*-ALLO binds in the opposite orientation from ALLO in the β_3_-α_1_ intersubunit site is consistent with our previous observation that mutations in the intersubunit binding site that interfere with neurosteroid action change steroid orientation in the binding site. In a wild-type ELIC-α_1_ GABA_A_ chimeric receptor, KK200 (photolabeling moiety on the D-ring) labeled residue Y309 at the bottom of TM3, whereas in receptors with the Q242L or W246L mutations, it labeled residue F298 in the middle of the TM3 helix [30]. These data indicate that the 3-hydroxy group on the steroid A-ring is oriented to the center of the transmembrane domain in wild-type receptors, but to the cytoplasmic end of the transmembrane helices in the mutant receptors. Elimination of a hydrogen bonding interaction between the 3-hydroxy group of a neurosteroid and the Q242 and/or W246 residues, either because of mutation or steroid structure, may favor a steroid orientation in which the 3-hydroxyl group points to the cytoplasmic interface with the membrane, similar to its preferred orientation in a lipid bilayer [31].

The results of this study elucidate the molecular interactions underlying ALLO enantioselectivity, but may also have some practical pharmacological implications. ALLO interacts with three binding sites on α_1_β_3_ GABA_A_ receptors, all of which mediate allosteric effects on channel function [9,15,18]. The preferential interaction of *ent*-ALLO with the intrasubunit sites suggests its potential utility as a site-selective ligand or as a scaffold for a site-selective neurosteroid ligand.

The enantiomers of ALLO were originally used to demonstrate that PAM neurosteroids act by binding to specific sites on the GABA_A_ receptor, rather than by perturbing the lipid membrane milieu in which the receptor resides [5]. Consistent with this idea, our data demonstrate differential binding of ALLO and *ent*-ALLO to the same site on a GABA_A_ receptor. However, our data for PREG and *ent*-PREG show that an enantiomeric pair of ligands can also bind to the same site with near identical affinity and efficacy. Thus, the presence of enantioselectivity supports a direct protein binding interaction, but its absence does not refute it. There is an old pharmacologic “rule” (Pfeiffer’s Rule) that states that the ratio (eudismic ratio) of the potency of a ligand (eutomer) to its less active enantiomer (distomer) is proportional to the potency of the eutomer [32]. ALLO and PREG bind with similar affinity to the β_3_/α_1_ intersubunit site on GABA_A_ receptors, producing a PAM effect of similar magnitude. However, *ent*-ALLO binds to the intersubunit site with much lower affinity than *ent*-PREG, producing a markedly smaller PAM effect. The difference in eudismic ratio between the ALLO and PREG enantiomeric pairs clearly contradicts Pfeiffer’s rule [33].

## Figures and Tables

**Table 1:** Lowest energy Vina scores for docking of steroids in the β_3_/α_1_ intersubunit neurosteroid binding pocket of the GABA_A_ receptor using the Cryo-EM structure of an α_1_β_3_γ_2_ receptor (PDB: 6HUO). Orientation 1 refers to poses in which the 3-hydroxy group of the steroid is oriented toward α_1_(Q242) and the methyl ketone is oriented toward β_3_(F301). Orientation 2 refers to poses in which the 3-hydroxy group is oriented toward β_3_(F301) and the methyl ketone is oriented toward α_1_(Q242). Docking was performed using AutoDock Vina embedded in UCSF Chimera ver. 1.16.

Ligand	Docking Score (kcal/mol.)	
	Orientation 1	Orientation 2
ALLO	−8.3	−7.4
*ent*-ALLO	−7.3	−7.9
PREG	−8.3	−7.5
*ent*-PREG	−7.4	−7.3

## Data Availability

The data presented in this study are available on request from the corresponding author.

## Supplementary Materials

The following are available online at https://www.mdpi.com/article/10.3390/biom13020341/s1, Figure S1: Fragmentation ion spectra of KK200 and KK123 labeled peptides. Figure S2: Mass spectrometric sequence coverage of α_1_ and β_3_ GABA_A_ receptor subunits.
