# Supplementary material for: The Mechanism of Enantioselective Neurosteroid Actions on GABAA Receptors
Source: Biomolecules. 2023 Feb 9;13(2):341. doi: 10.3390/biom13020341 (PMC9953308; doi:10.3390/biom13020341)
Supplement: Supplementary file 1 [file biomolecules-13-00341-s001.zip › biomolecules-2186433-supplementary.pdf]

Supplementary Materials

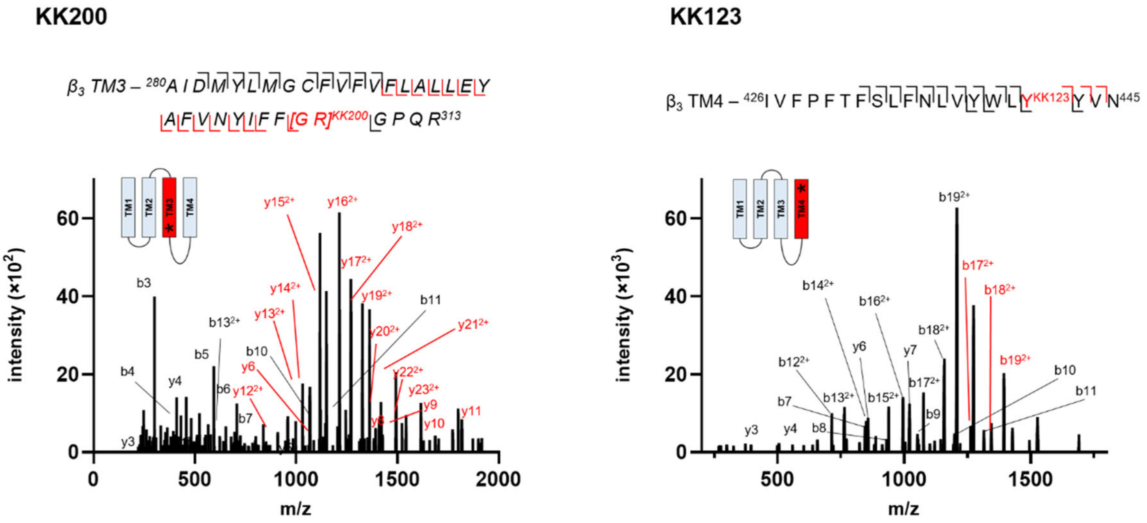

Figure S1. Fragmentation ion spectra identifying GABAA receptor residues photolabeled by KK200 and KK123. **Left.** HCD fragmentation spectrum of the β3-subunit TM3 peptide photolabeled by 30 μM KK200. Red and black indicate fragment ions that do or do not contain KK200, respectively. The schematic highlight in red identifies the TMD being analyzed and the asterisk denotes the approximate location of the KK200 labeled residue. **Right.** HCD fragmentation spectrum of the β3-subunit TM4 peptide photolabeled by 30 μM KK123. Red and black indicate fragment ions that do or do not contain KK123, respectively.

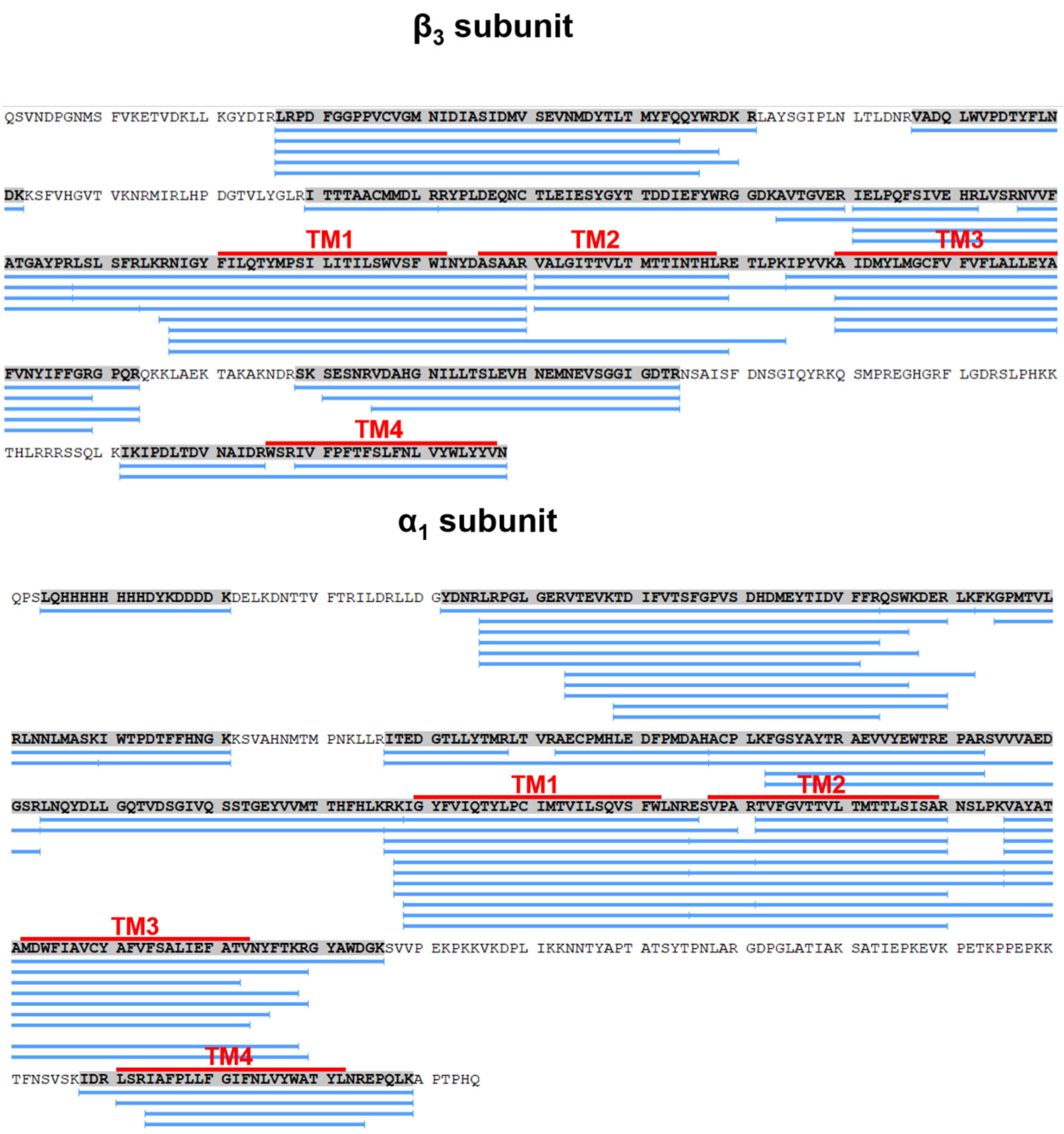

Figure S2. Mass spectrometric sequence coverage of α_1 and β_3 subunits of the $GABA_A$ receptor based on analysis of HCD fragmentation ion spectra. Blue lines indicate identified peptides. Red lines indicate the transmembrane domains (TM1-4). 100% sequence coverage was obtained for the transmembrane domains of both the α_1 and β_3 subunits. The digestion conditions and chromatographic separation are designed to detect the larger hydrophobic TM1-4 domains. Hydrophilic peptides and smaller tryptic peptides characteristic of the extracellular and intracellular domains are not reliably detected using this approach.
